# Ilizarov-Assisted Healing for a Neglected Non-united Fracture Calcaneus: A Case Report and Literature Review

**DOI:** 10.7759/cureus.57011

**Published:** 2024-03-27

**Authors:** Mohamed A A Ibrahim, Usama Gaber, Mostafa M Elgahel, Samir A Nematallah

**Affiliations:** 1 Orthopedic Department, Faculty of Medicine, Al-Azhar University, Cairo, EGY

**Keywords:** fracture calcaneus, neglected, nonunion, ilizarov frame, foot and ankle

## Abstract

Although calcaneal fracture is not a rare injury and nonunion is rare, proper management of a calcaneal fracture is mandatory because it can be a prerequisite for long-term functional disabilities of the foot, including posttraumatic osteoarthritis of the hindfoot joint, chronic pain, and persistent swelling syndromes. Restoration of axial alignment and joint congruence with careful caution toward soft tissues is the basic principle of treatment; however, few literature reviews to date have addressed the characteristics of a calcaneal nonunion fracture.

We discuss a case of a 30-year-old male, manual worker, and diabetic type 1 with a calcaneal fracture reaching the articular surface of the subtalar joint who underwent a simple fracture to a painful nonunion fracture after conservative treatment for seven months before presenting to our hospital being unable to walk with heel deformity. The Ilizarov frame was used to correct deformities in the hindfoot, enhance healing by compressing the fracture site, and allow early weight bearing with the maintenance of subtalar joint function.

Our result demonstrates increased calcaneal healing when the Ilizarov foot frame is used, and when the calcaneal fracture site is compressed, this is a good option for maintaining foot and ankle function, even in diabetic patients.

## Introduction

Physically fit and active males are the most vulnerable to calcaneal fractures [1]. According to Essex-Lopresti, 75-92% of calcaneal fractures involve a fracture line that runs through the articular surface and are classified as intra-articular fractures with an impression-shifting nature. Proper management of a calcaneal fracture is mandatory because it can be a prerequisite for long-term functional disabilities of the foot, including posttraumatic osteoarthritis of the hindfoot joint, chronic pain, and persistent swelling syndromes [2].

The uncommon consequences of calcaneal nonunion following management have been mentioned in only a small number of case reports and their successful management. Restoration of axial alignment and joint congruence, with careful caution toward soft tissues, is the basic principle of treatment [1].

## Case presentation

**Figure 1:**
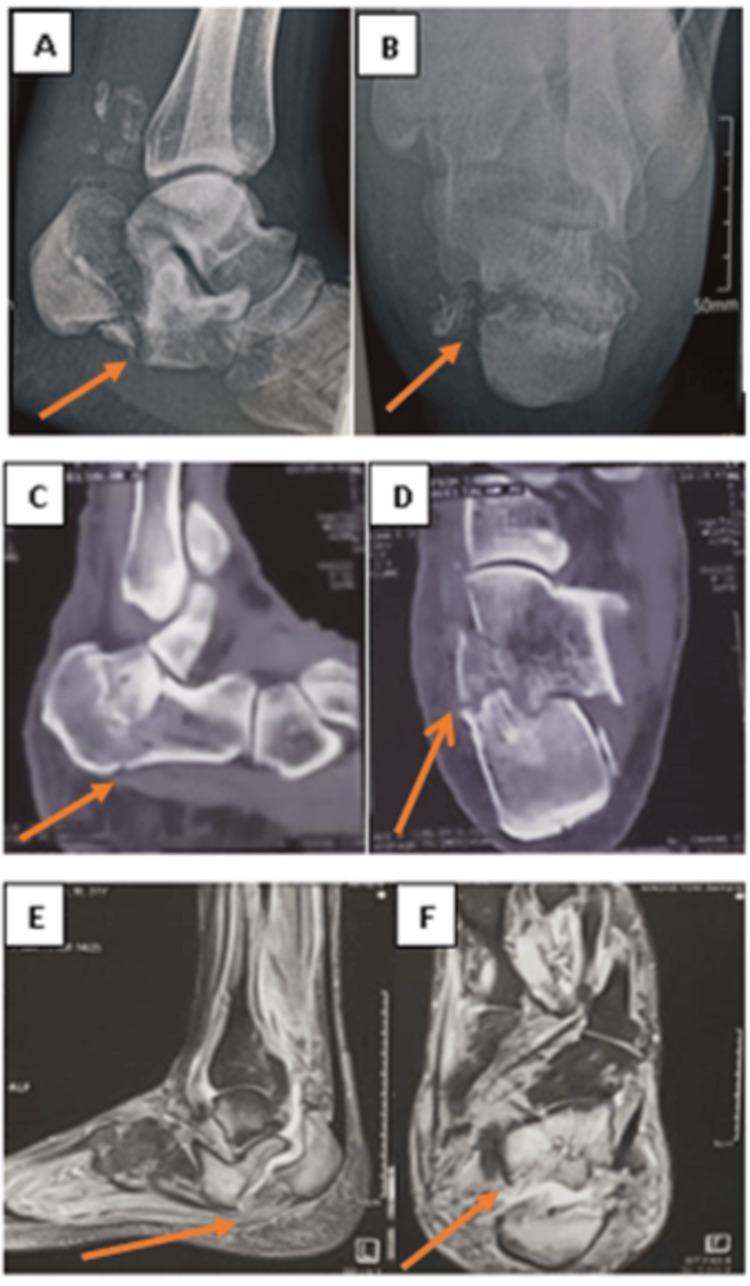
Preoperative images of the fracture included. Plain X-ray: (A) lateral view and (B) axial view. CT scan: (C) sagittal view and (D) semi coronal view. MR images: (E) sagittal view and (F) axial view

A 30-year-old male manual worker who fell from 1.5 meters, had diabetic type 1 (20 years ago), sustained a calcaneal Sanders type II fracture, and underwent conservative treatment for seven months presents to our hospital with complaints of being unable to walk. The clinical evaluation shows heel deformity in the form of equinus, varus, and broadening, and radiological assessment, including a CT scan and MRI of the foot, shows calcaneal nonunion fracture (Figure 1).

Surgical technique (closed percutaneous method)

**Figure 2:**
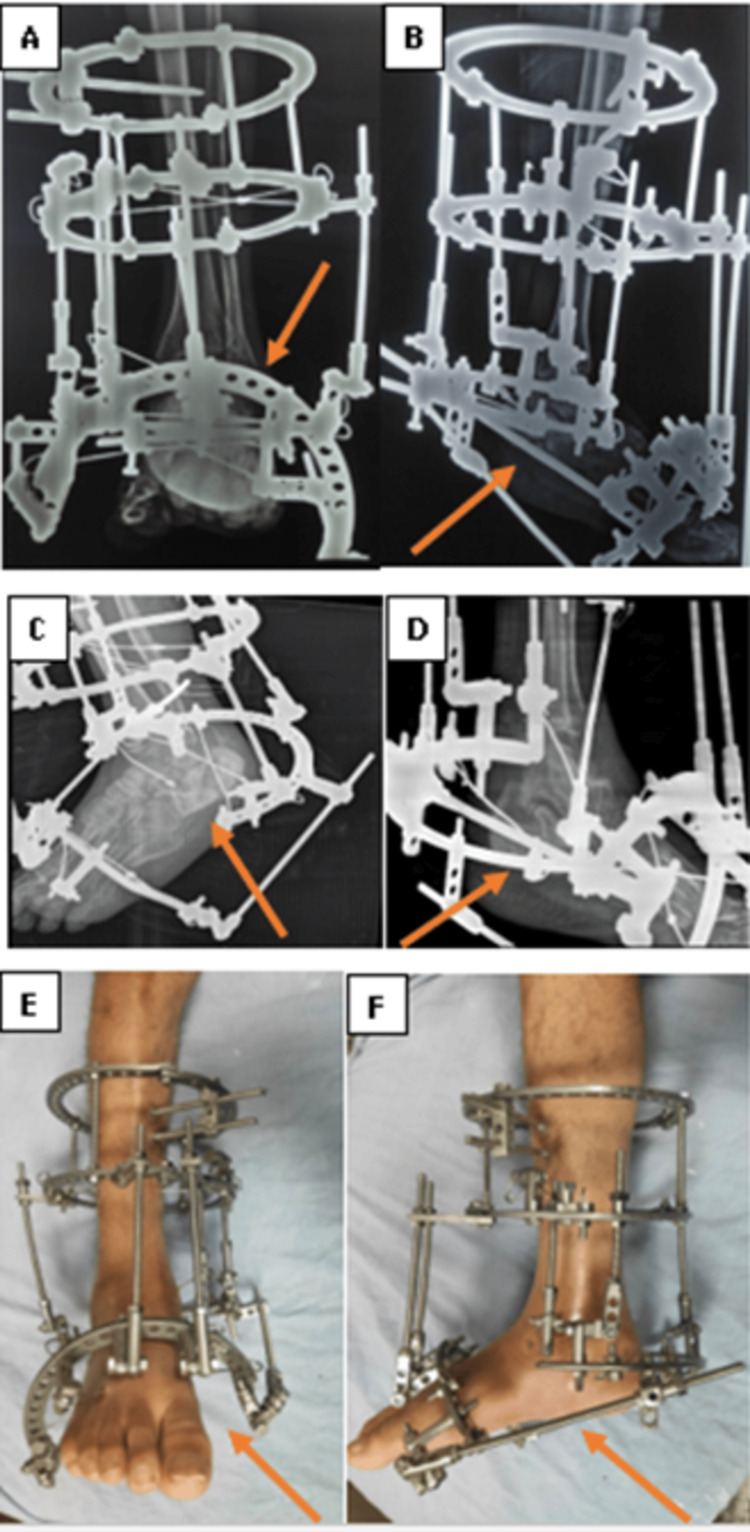
(A, B, C, D) Immediately postoperative plain X-ray. (E, F) Clinical image of the patient frame

Under complete aseptic conditions, the Ilizarov frame is composed of the proximal block in the tibia and the distal block in the calcaneus and the forefoot; the proximal block is composed of two rings of equal size connected by four rods stabilized by two Schanz pins and a bayonet wire. The olive trans-fibular-tibial wire, bayonet wire, and Schanz pin were used to stabilize the supra-malleolar ring. The distal block is made up of a half-ring in the calcaneus and a half-ring in the metatarsal forefoot. Two olive wires cross each other, and the Schanz pin serves to stabilize the half ring in the calcaneus. The half-ring in the forefoot of the metatarsal was stabilized by two intersecting olive wires. One wire and Schanz pin were used to stabilize the talus before being linked to the proximal block (Figure 2).

**Figure 3:**
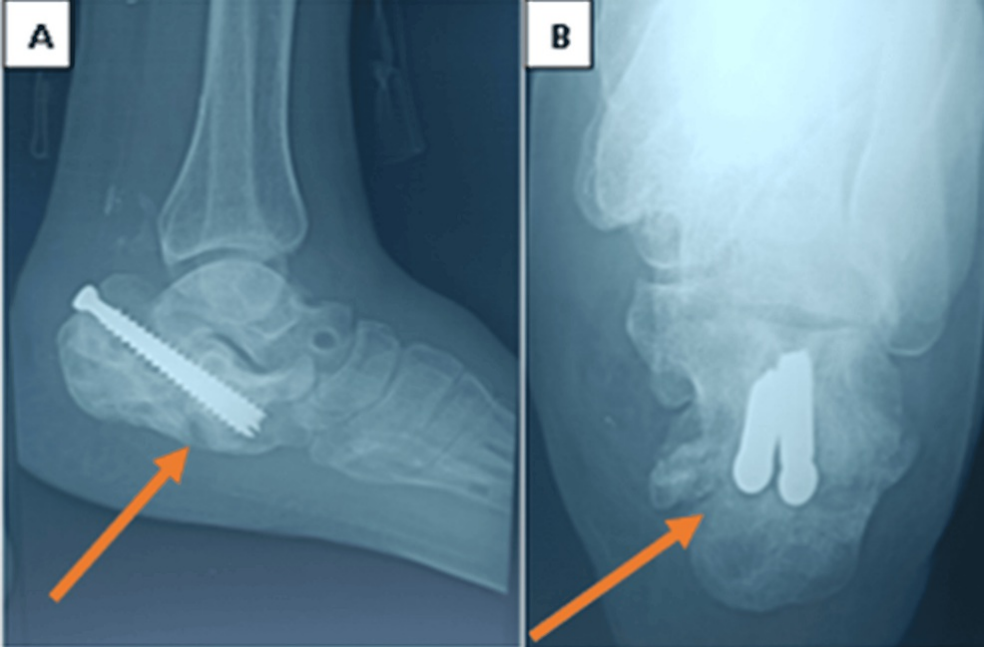
Follow-up plain X-ray: (A) lateral view and (B) axial view. After frame removal, a complete union of the fracture was observed

**Figure 4:**
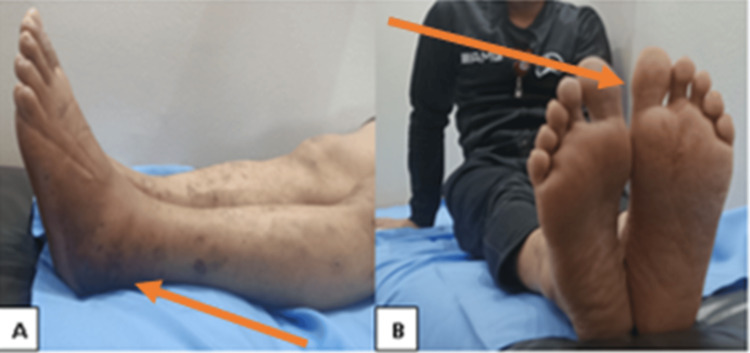
A clinical follow-up image of the patient after the removal of the frame shows the correction of the hindfoot deformities and a good range of motion of the ankle and foot

The leg support and foot component are attached together, rods and biplanar hinges are used to attach the leg support and calcaneal component. Rods and biplanar hinges are used to connect the foot component to the calcaneal component. The correction of equinus due to collapse of the calcaneus bone and varus deformities was achieved by lengthening the posterior rod between the leg support and the calcaneal component, lengthening the medial rod, and shortening the lateral rod. Additionally, two threaded rods were positioned between the calcaneal half ring and the forefoot. Compression by shortening was performed after deformity repair at the nonunion location between the forefoot and the calcaneal half-ring. After three months, we removed the frame after the achievement of union and secured it with two cancellous screws (Figures 3-4).

## Discussion

The long-term effects of calcaneal fractures have demonstrated their difficulty in managing them and the multitude of complications they might cause, including subtalar osteoarthritis and malunion. A very uncommon consequence that has been documented in case reports in the literature is the nonunion of the calcaneum [3,4]. It is possible to view conservative treatment as a risk factor for nonunion, as demonstrated by the observation of Thermann et al. that 10% of their series experienced nonunion following conservative treatment [5].

Because of infrequent reporting, nonunion following a calcaneal fracture may be underestimated. Only a few case reports published in 11 research studies make up the present body of literature in English language only (Table 1).

**Table 1:** Case reports of calcaneal nonunion in literature and their methods of treatment

Study	Number of patients	Treatment of nonunion	Follow-up (month)
Sherehii et al. [1]	1	Plate, bone graft	6
Wajdi et al. [3]	1	Bone marrow concentrates	12
Zhang et al. [4]	2	Subtalar arthrodesis, conservative	11,14
Thermann et al. [5]	1	Subtalar arthrodesis	62
Thomas and Wilson [6]	1	Osteotomy, plate, bone graft	3
Gehr et al. [7]	1	Osteotomy, screws, bone graft	2
Karakurt et al. [8]	1	Bone graft	8
Zwipp and Rammelt [9]	2	Subtalar arthrodesis	-
Schepers and Patka [10]	3	Subtalar arthrodesis	3,14,6
Kumar [11]	1	Subtalar arthrodesis, screws, bone graft	12
Molloy et al. [12]	14 (15 nonunions)	Subtalar fusion, triple arthrodesis, calcaneal osteotomy, bone graft	72 (24-102)
Present study	1	Compression by Ilizarov	3

Currently, when treating calcaneal fractures, orthopedic surgeons should be aware of the risk factors and implement preventative measures. Patients were advised to regulate their blood sugar levels and to give up smoking. Patients who have experienced significant trauma should receive additional care. A carefully planned surgical plan with thorough and sufficient preoperative communication is essential if nonunion is present [4].

Arthrodesis is currently the main surgical procedure used to address calcaneal nonunion. Given the intricacy of the procedure, the low quality of the bone, and the severity of the abnormalities, it might be necessary to perform many revision surgeries with potential postoperative consequences [13].

Distraction histogenesis principles are a technological solution in which, regardless of how effectively the subtalar joint architecture is repaired, the height and length of the bone are quickly restored, which is a predictor of good long-term outcomes in the reduction of displaced calcaneal fractures. Next, to promote healing, compression is applied at the fracture site.

There are no similarities, as far as we know, between ring fixators and nonunion management. The fundamental ideas and methods of closed fracture reduction, stabilization, and compression are applied in the external ring fixation treatment of nonunion calcaneal fractures using tensioned wires fastened to the frame. The ability to divert the calcaneus at the subtalar joint while the frame is in place is another special characteristic of external ring fixation, which helps to restore the height and length of the calcaneus bone.

The limitations of this technique include the experience of the surgeon in dealing with calcaneal fractures, and to avoid the calcaneus from misaligning the varus or valgus, skeletal traction should be administered gently to the extremities in the sagittal plane. This may negatively impact future ankle and subtalar joint function.

Our technique is a good substitute for treating nonunion calcaneal fractures since it accomplishes the objectives of restoring calcaneal length, width, height, and subtalar joint preservation surgery through distraction and fracture site compression.

One of the most common surgical treatment complications for patients who have undergone calcaneal fracture surgery is wound issues. Our method provides a secure substitute that can be applied in these circumstances. Using the Ilizarov external fixation system and deformity correction principles, a minimally invasive approach is used to reduce and compress calcaneal fractures. No metal hardware should be left inside the foot.

## Conclusions

We are presenting a case of a diabetic patient with neglected calcaneal nonunion, which led to hindfoot deformity and affected his walking ability, for whom surgical treatment by Ilizarov frame was performed. Our result demonstrates increased calcaneal healing when the Ilizarov foot frame is used and when the calcaneal fracture site is compressed. This is a good option for maintaining foot and ankle function, even in diabetic patients.
